# A case of gangliocytic paraganglioma in the ampulla of Vater

**DOI:** 10.1186/1477-7819-8-42

**Published:** 2010-05-24

**Authors:** Junsik Kwon, Seung Eun Lee, Mee Joo Kang, Jin-Young Jang, Sun-Whe Kim

**Affiliations:** 1 Department of Surgery, Seoul National University College of Medicine, Seoul, Korea

## Abstract

**Background:**

Duodenal gangliocytic paraganglioma is an extremely rare tumor and few cases have been reported to date.

**Case presentation:**

The authors report a case of gangliocytic paraganglioma verified by post-op pathology after pancreaticoduodenectomy for a tumor in the ampulla of Vater. The 56-year-old male patient concerned visited our emergency room with melena that started one week prior to hospitalization. The patient was diagnosed to have a tumor in the ampulla of Vater with bleeding on its surface. However post-op, he was diagnosed as having gangliocytic paraganglioma by immunohistochemistry.

**Conclusion:**

This tumor has precise clinical implications, and if continuous follow up is conducted after careful diagnosis and surgical treatment, invasive major operations, such as, radical pancreaticoduodenectomy can be avoided.

## Background

Gangliocytic paragangliomas are rare tumors which are usually encountered in the second portion of the duodenum. They can be diagnosed histologically by the presence of epithelioid, spindle, and ganglion cells, which is similar to that observed for paraganglioma [1]. Although gancliocytic paragangliomas have no specific accompanying symptoms, they are sometimes found due to bleeding caused by mucosal ulceration, and rarely because of huge mass effect, such as, abdominal pain or obstruction. However, they are usually detected incidentally during radiologic imaging conducted for different purposes [2]. Here, we report a gangliocytic paraganglioma in the second portion of the duodenum in a patient hospitalized for melena, which was removed by pancreaticoduodenectomy. We also include a review of the literature.

## Case presentation

**Figure 1:**
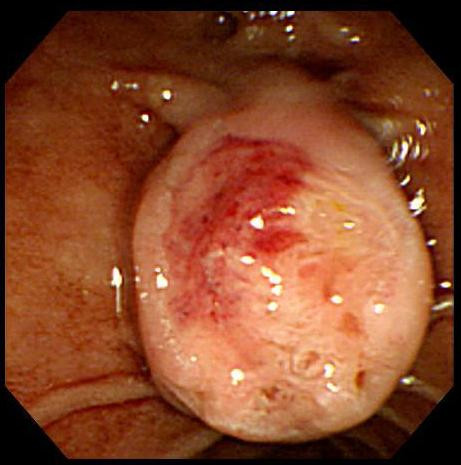
**Esophagogastroduodenoscopic findings showing the periampullary submucosal tumor and surface ulcer bleeding**.

**Figure 2:**
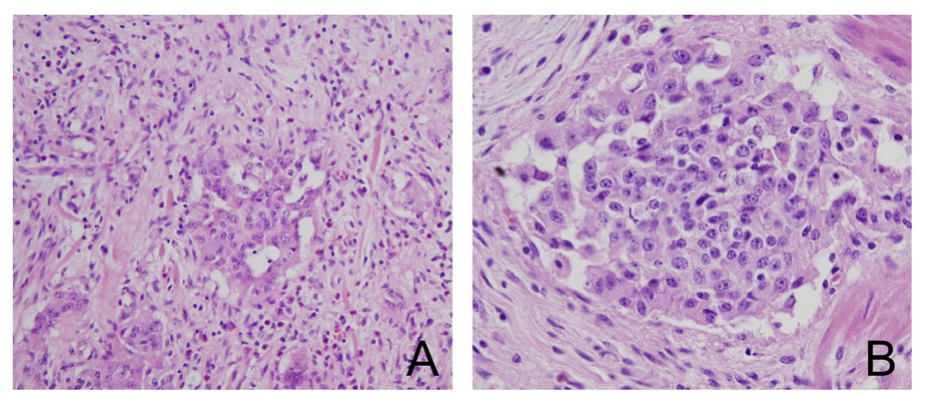
**(A) The mass lesion showed a triphasic pattern comprised of epithelioid cell nests, neurofibromatous spindle cells and ganglion cells**. (×200) (B) Carcinoid-like epithelioid cell nests with surrounding spindle cells. (×400).

**Figure 3:**
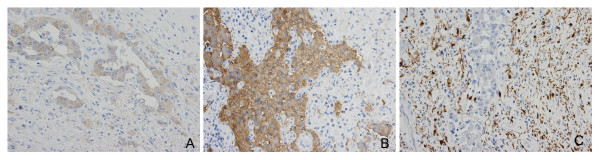
**Immunohistochemical stains for (A) neuron-specific enolase, (B) synaptophysin, and (C) S-100: Epithelioid cell components were positive for neuron-specific enolase and synaptophysin, and neurofibromatous spindle cell components were positive for S-100**.

A 56-year-old male patient visited our emergency room due to melena of duration one week. History taking revealed no particular issues other than antihypertensive medication after a diagnosis of hypertension five years previously. He did no smoke, but consumed a small amount of alcohol regularly. No specific features arose from his family or social history. He did not experience nausea or vomiting at the time of hospitalization, and only complained of mild indigestion. Furthermore, he showed no epigastric soreness, abdominal pain, or weight loss, and his vital signs at hospitalization were stable. His physical examination was uneventful. His hemoglobin was 10.4 g/dL, and renal and liver function, as determined by blood tests, were also normal. No lesions were found in the esophagus or stomach by esophagogastroduodenoscopy. However, an exophytic tumor with a bleeding surface ulcer was observed luminally in the ampulla of Vater in the second portion of the duodenum (Figure 1). An endoscopic biopsy was performed on the tumor and bleeding from the ulcer was controlled endoscopically. And abdominal computer tomography (CT) and magnetic resonance imaging (MRI) revealed a hypoattenuating mass of diameter 1.6 cm in the second portion of the duodenum. The pathological result later revealed atypical chronic inflammation and regenerative atypia. Although no malignant cells were observed, surgery was performed based on the judgment that gross findings indicated that the possibility of malignancy was high. During surgery, a papillary 2.5 × 2.0 × 0.7 cm sized mass was found in the ampulla of Vater. Distant metastasis or any of lymph node enlargement were not observed. Pylorus preserving pancreaticoduodenectomy (PPPD) was performed. The pathological result of the excised specimen showed that the tumor was limited to the mucosa and proper muscle layer and had not invaded the pancreas or common bile duct. Furthermore, no lymph node metastasis was detected. The submucosal tumor was found to have a triphasic pattern in low power fields, whereas high power fields showed that the tumor was composed of nests of endocrine cell and ganglion cells with abundant cytoplasm, and spindle cells were found to surround tumor cells (Figures 2A and 2B). Immunohistochemistry showed that tumor cells were positive for synaptophysin, neuron specific antigen, and S-100. In addition, focal positive responses were observed for chromogranin, but no cytokeratin response was observed (Figures 3-A, B, C). Based on the above features, the mass was diagnosed as a gangliocytic paraganglioma. During on-going regular follow-up visits no evidence of recurrence or metastasis was observed from December 2007 to April 2009.

## Discussion

Gangliocytic paraganglioma is a rare benign tumor of the digestive tract. Although some have reported cases of gangliocytic paraganglioma invading the proximal jejunum, about 90% are found in the second part of the duodenum, from where the tumor can invade the ampulla of Vater[3]. In the WHO classification of tumors of digestive tract (2000), gangliocytic paraganglioma was independently classified as a type of epithelial tumor. Other duodenal neuroendocrine tumors, except for non-differentiated neuroendocrine carcinoma, were classified as carcinoid tumors[4]. Males are affected slightly more commonly than females (1 to 1.8/1) and in terms of age at onset although the fifties are preferred, it has been encountered over an age range from 23 to 83 years[5]. The endoscopic features of gangliocytic paraganglioma do not differ from those of other submucosal tumors. However, its preoperative pathologic diagnosis is difficult based on endoscopic biopsy alone, because of its submucosal nature, and therefore, endoscopy must be assisted by radioscopy. Gangliocytic paraganglioma is well defined by ultrasonography and is visualized as an isoechoic mass, whereas abdominal computer tomography visualizes it as mass-like soft tissue that is homogenously iso-attenuated, as is observed in muscles beside the vertebrae [6]. Pancreatic head cancer in the duodenum, duodenal cancer, duodenal sarcoma, angioma, choledochal cyst, lipoma, hamartoma, and lymphoma must be differentiated from gangliocytic paraganglioma by radiography. This differentiation can be performed based on lesion's location, degree of attenuation by abdominal computer tomography, CBD dilatation, and enhancing pattern [7]. However, accurate preoperative diagnosis is often difficult, due to the lack of histological confirmation. Although gangliocytic paraganglioma is incidentally found by radiological examinations and is asymptomatic in most cases, symptoms when present may be location dependent. According to a review of 51 cases reported in the literature by Burke et al [5], the reported symptoms were; abdominal pain in 13 cases, gastrointestinal bleeding in 6, melena in 6, anemia in 5, pyloric obstruction in one, and bile duct obstruction in one. Gangliocytic paraganglioma is histologically composed of three cell types, namely, epithelioid, ganglion, and spindle cells. However, the compositions of these cells vary [8]. Nevertheless, gangliocytic paraganglioma is verifiable by immunohistochemical examination. In the described case, the patient was positive for synaptophysin and neuron specific antigen, focally positive for chromogranin, partially positive in stroma for S-100, and negative for cytokeratin. Several authors have reported that epithelioid and ganglion cells are positive to neuroendocrine peptides, such as, somatostatin, pancreatic polypeptide, and serotonin. Furthermore, it has been argued that epithelioid cells have the same origin as ganglion cells and that they are related to islet cell tumors [9], or alternatively, that carcinoid tumors of the duodenum have the proliferative growth pattern or harmatoma-like growth characteristics of carcinoid tumors [10]. Gangliocytic paragangliomas follow a benign course and invasive growth patterns and lymph node metastasis are rare even for large tumors [11]. Furthermore, in few cases with regional lymph node metastasis distant metastasis was not observed [5,12]. Though lymph node metastasis usually only involves the transfer of epithelioid cells [12], in one case report all three cell types were transferred [13]. Furthermore, although recurrence is generally considered not to occur, there are rare reports of gangliocytic paraganglioma recurrence [13,14]. Tumors of the duodenum often require pancreaticoduodenectomy or lymph node dissection. However, because metastasis and the recurrence of gangliocytic paraganglioma is rare, and moreover no case of death resulting from this tumor has been reported, mass excision is considered sufficient to treat as long as abnormal features are not found in lymph nodes, and bile and pancreatic ducts by endoscopic ultrasonography. As was performed in our case, radical excision including pancreaticoduodenectomy has usually been performed, although reports are emerging regarding endoscopic resection [9]. However, because the possibilities of recurrence and metastasis cannot be completely excluded, decisions on treatment methods must be made after careful preoperative staging of the disease prior to local treatment [12]. Furthermore, continuous follow up at the out-patient department for early detecting of recurrence is deemed necessary.

## Conclusion

Here we report a case of gangliocytic paraganglioma in the ampulla of vater. Although gangliocytic paraganglioma in the duodenum is an extremely rare disease, it shows a good prognosis as compared with other peri-ampulla of Vater tumors. Furthermore, if continuous follow up observation is conducted after obtaining a careful diagnosis, it can be treated only limited surgery like local excision, without performing pancreaticoduodenectomy or lymph node dissection.

## Competing interests

The authors declare that they have no competing interests.

## Authors' contributions

KJS conceptualized the study, gathered the data, and drafted the manuscript, LSE performed the literature search and helped to draft the manuscript, JJY supervised the process and finally approved the manuscript for publication, KMJ and KSW was involved in manuscript revision. All authors have read and approved the final manuscript.

## Consent

Written informed consent was obtained from patient for reporting of this case, the copy of consent is available with editor in chief.
